# Distribution of Usutu Virus in Germany and Its Effect on Breeding Bird Populations

**DOI:** 10.3201/eid2312.171257

**Published:** 2017-12

**Authors:** Renke Lühken, Hanna Jöst, Daniel Cadar, Stephanie Margarete Thomas, Stefan Bosch, Egbert Tannich, Norbert Becker, Ute Ziegler, Lars Lachmann, Jonas Schmidt-Chanasit

**Affiliations:** Bernhard Nocht Institute for Tropical Medicine, World Health Organization Collaborating Centre for Arbovirus and Hemorrhagic Fever Reference and Research, Hamburg, Germany (R. Lühken, H. Jöst, D. Cadar, E. Tannich, J. Schmidt-Chanasit);; German Centre for Infection Research (DZIF), partner site Hamburg-Luebeck-Borstel, Hamburg (H. Jöst, E. Tannich, J. Schmidt-Chanasit);; University of Bayreuth, Bayreuth, Germany (S.M. Thomas);; Nature and Biodiversity Conservation Union (NABU), Stuttgart, Germany (S. Bosch);; Institute for Dipterology, Speyer, Germany (N. Becker);; University of Heidelberg, Heidelberg, Germany (N. Becker);; Friedrich-Loeffler-Institut, Greifswald-Insel Riems, Germany (U. Ziegler);; Nature and Biodiversity Conservation Union (NABU), Berlin, Germany (L. Lachmann)

**Keywords:** Usutu virus, flavivirus, breeding birds, common blackbird, bird population decline, vector-borne infections, Germany

## Abstract

Usutu virus (USUV) is an emerging mosquitoborne flavivirus with an increasing number of reports from several countries in Europe, where USUV infection has caused high avian mortality rates. However, 20 years after the first observed outbreak of USUV in Europe, there is still no reliable assessment of the large-scale impact of USUV outbreaks on bird populations. In this study, we identified the areas suitable for USUV circulation in Germany and analyzed the effects of USUV on breeding bird populations. We calculated the USUV-associated additional decline of common blackbird (*Turdus merula*) populations as 15.7% inside USUV-suitable areas but found no significant effect for the other 14 common bird species investigated. Our results show that the emergence of USUV is a further threat for birds in Europe and that the large-scale impact on population levels, at least for common blackbirds, must be considered.

Usutu virus (USUV) is a mosquito-borne flavivirus that, together with West Nile virus (WNV), belongs to the Japanese encephalitis antigenic complex (*1*). Both viruses share a similar enzootic transmission cycle, with birds as amplifying hosts and ornithophilic mosquitoes as vectors (*2*). Mammals, including bats, horses, and humans, are considered incidental or dead-end hosts (*3**–**6*).

The clinical picture of human USUV infection includes fever, rash, jaundice, headache, nuchal rigidity, hand tremor, and hyperreflexia (*7*). It has been generally assumed that the incidence of human USUV infections is very low compared with the incidence of WNV infections. However, this assumption is probably strongly biased by the comparatively low capacity to correctly identify USUV infection in humans (*2**,**8*). Recent data from Italy indicate that human USUV infections may not be a sporadic event and can even be more frequent than WNV infections in areas where both viruses co-circulate (*9*).

The most common recent ancestor of the USUV strains circulating in Europe emerged in Africa at least 500 years ago (*10*). In 1996, the first recognized USUV outbreak outside Africa caused a massive die-off among common blackbirds (*Turdus merula*) in the Tuscany region of Italy (*11*). During the next 2 decades, USUV was observed in several countries in Europe as responsible for periodic small epizootic outbreaks affecting birds (*2**,**12*). In Germany, the earliest observation of USUV was in 2010 in mosquitoes (*13*) and resulted in mass deaths of common blackbirds (*12**,**14*) and at least 2 human USUV infections (*4**,**6*).

Mosquitoborne pathogens such as WNV (*15*) or avian malaria (*16*) could have substantial negative effects on bird populations, such as the size of affected populations (*15*) or composition of species communities (*16*). Several bird taxa of different taxonomic orders were found to be susceptible to USUV infections (*2*); during the USUV outbreaks in Europe, common blackbirds accounted for the largest proportion of observed dead birds (*2*). The effect of USUV on the populations of this species is discussed in different studies, which gave different estimates for population declines. Savini et al. (*17*) estimated l,000 deaths for blackbirds in Veneto, Italy, in 2008–2009. During the USUV outbreak in Vienna, Austria, and surrounding areas in 2003–2005, Steiner and Holzer (*18*) observed a population decline of ≈90%. Furthermore, Rubel et al. (*19*) estimated that only 0.2% of all dead blackbirds were detected by the national USUV monitoring program in Austria (*20*), and therefore, ≈50,000 birds died during the outbreak. Using the same method of estimation, ≈40,000 common blackbirds died in the earliest known outbreak (2011–2012) in Germany (*21*). Further studies in Germany estimated 220,000–420,000 dead common blackbirds (*22*) and a local population reduction of >50% (*23*). 

The existing studies lack 2 conditions: an explicit spatial distinction between areas in which USUV circulates and those in which it does not; and the analysis of long-term bird population data, which are necessary to test the hypotheses that USUV caused substantial population declines in birds. Therefore, 20 years after the first observed outbreak of USUV in Europe (*11*), a reliable assessment of the large-scale impact of USUV outbreaks on bird populations is missing. Identifying the population-level effects of the disease is challenging, because they must be distinguished from natural population fluctuations driven by environmental factors such as climate (*15*) or land use change (*24*). Thus, the analyses require long-term bird abundance data that extend before and after the emergence of the disease and that cover areas with and without circulation of the pathogen. Hence, the aims of this study were the identification of areas suitable for USUV circulation in Germany using a distribution modeling approach based on dead bird surveillance data and the comparative analysis of USUV effects on the breeding bird populations in USUV-suitable areas.

## Materials and Methods

### Distribution Modeling Data

The USUV data in Germany were collected as part of a dead bird surveillance program (*12**,**14**,**25**,**26*). After the description of a USUV outbreak in wild birds in Germany in 2011, we requested, by press releases and media dissemination, that citizens send dead birds for USUV screening at national reference laboratories. We necropsied the bird specimens and screened them for USUV-specific RNA. During August 2011–November 2015, a total of 230 specimens of 15 species (85.7% common blackbirds) from 132 different sites tested positive for USUV. We used European Land Surface Temperature (EuroLST) dataset maps with 9 bioclimatic variables at 250-m resolution as explanatory variables for the distribution modeling of USUV (*27*). Bioclimatic variables are derived from monthly temperature and rainfall values. These biologically meaningful variables represent annual trends, seasonality, and extreme or limiting environmental factors.

### USUV Distribution Modeling

We applied an ensemble boosted regression tree (BRT) approach using R software (https://www.r-project.org) with the packages raster, dismo, and ecospat and visualized with ggplot2, which was successfully applied to other mosquitoborne viruses in the past (e.g., Zika virus) (*28*). We calibrated BRT models with presence-only data and 10,000 random background points selected from the entire area of Germany. To account for the biased bird collection due to the unsystematic dead bird surveillance program and to increase the robustness of model predictions and quantify model uncertainty, we selected 300 random subsamples of the presence data with replacement. Due to locality uncertainties in the presence data (e.g., mobility of the birds and imprecise reporting by the volunteer senders), we applied a random point selection within the corresponding German postal code areas (0.31–891.68 km^2^, mean size 32.00 km^2^) for all presence points (i.e., sites with birds testing positive for USUV) in each subsample. In addition, we selected a new set of 10,000 random background points for each model. We weighted background points and occurrence points equally in each of the 300 BRT models, which we averaged for the final USUV distribution map. We converted the continuous distribution map for USUV to a binary map with areas that are suitable or unsuitable for USUV. Following Pigott et al. (*29*), we selected a threshold that included 90% of the USUV occurrence points. We chose a threshold cutoff of 90% instead of 100% to account for potential spatial inaccuracies of the occurrence point dataset.

We validated the models with a 10-fold cross-validation approach. We produced a total of 300 random split sampling datasets with 10 subsets for training datasets (comprising 10% of the presence and background observations) and 10 subsets for test datasets (comprising 90% of the presence and background observations) each. We used the training datasets to assess the ability of the models to predict the test dataset with the area under the curve (AUC) statistic. We averaged the AUC values of the models across the 10 models of each split sampling dataset and finally across the 300 average AUC values. Furthermore, we applied a pairwise distance sampling procedure to avoid AUC inflation due to spatial sorting bias, which is considered to give a more realistic quantification of the model performance especially regarding its transferability (*30*).

### Bird Population Data

Bird abundance data were collected within the citizen science program Stunde der Gartenvögel (Hour of the Garden Birds) in Germany. This program is organized by the German BirdLife partner Naturschutzbund Deutschland (Nature and Biodiversity Conservation Union) and its counterpart Landesbund für Vogelschutz in Bayern (Bavarian Society for the Protection of Birds). During the second weekend of May each year, German citizens were requested to count the maximum number of specimens per bird species observed in their gardens in a time frame of 1 hour. We used the data for the 15 most commonly detected bird species, with at least 247,000 observed specimens each during 2006–2016, for further analyses: Eurasian blue tit (*Cyanistes caeruleus*), common chaffinch (*Fringilla coelebs*), Eurasian magpie (*Pica pica*), European greenfinch (*Chloris chloris*), black redstart (*Phoenicurus ochruros*), great tit (*Parus major*), common blackbird, house sparrow (*Passer domesticus*), Eurasian tree sparrow (*Passer montanus*), common swift (*Apus apus*), common house martin (*Delichon urbicum*), carrion crow (*Corvus corone*), common wood pigeon (*Columba palumbus*), European robin (*Erithacus rubecula*), and common starling (*Sturnus vulgaris*). The dataset consisted of 317,533 unique observation datasets with anonymized sampling locations at the level of postal code regions in Germany, each giving information on the number of specimens per bird species and sampling site. 

### Bird Population Modeling

We applied a generalized additive model approach to analyze the population development of each of the 15 bird species. This statistical approach was first developed by Fewster et al. (*31*) to describe population trends in breeding birds and later successfully used to model bird and bat populations (*32**,**33*). We used the GAM framework to fit a single smoothed curve to the trend of the number of bird specimens in the USUV-positive areas and USUV-negative areas per year. In addition, to allow for differences in relative abundances between sites, we included a site term in the models. Following the suggestion by Fewster et al. (*31*), we set the selection of the degree of smoothing in the GAM to 0.3 times the number of years of the survey data (df = 3).

We avoided the problems of temporal autocorrelation within the abundance data (*31*) and overdispersion (*34*) by using a bootstrap approach. We produced CIs around the smoothed trends with a total of 300 bootstrap samples by resampling with replacement observations from the original dataset for each bird species. We classified each sampling site of the bird population data to be located in the USUV-positive or USUV-negative area on the basis of site coordinates and the USUV binary map. Spatial information of the bird observation sites was available only at the level of postal code regions; therefore, for each bootstrap sample, we took the mean of observed specimens per species within each region and then randomly assigned it within its postal code region to classify it as within the USUV-suitable or USUV-unsuitable area.

We set 2011 as the baseline year (index = 100); this year was the last time bird abundance data were collected before the first epizootic outbreak of USUV in Germany (*14*). Nonoverlapping 95% bootstrap CIs with index = 100 and nonoverlapping 95% bootstrap CIs between the USUV-suitable areas and USUV-unsuitable areas in 2016 were interpreted as a statistically significant difference (p<0.05).

## Results

**Figure 1:**
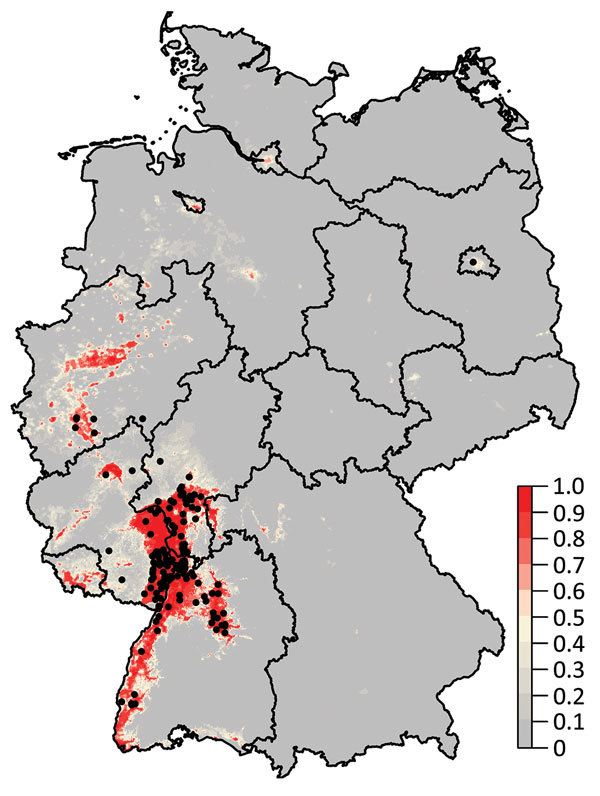
Probability of Usutu virus (USUV) occurrence in Germany derived from 300 boosted regression tree models. Black dots denote sites with dead birds that tested positive for USUV. The color intensity indicates the probability of occurrence of USUV.

**Figure 2:**
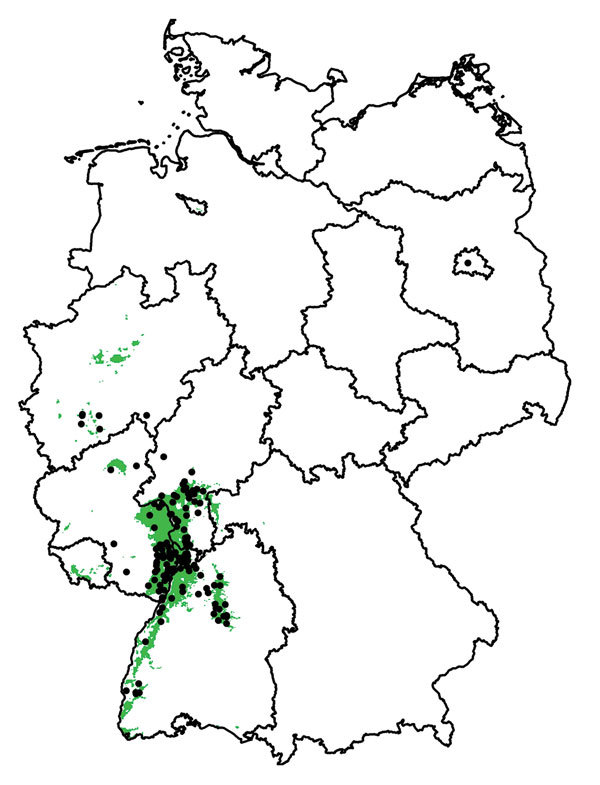
Areas suitable (green) and unsuitable (white) for Usutu virus (USUV) in Germany derived from 300 boosted regression tree models. Black dots denote sites with dead birds detected positive for USUV.

The mean of the ensemble of 300 BRTs indicated the highest probability for USUV circulation in southwestern Germany (Figures 1,2; Technical Appendix Figure 1). Environmentally suitable areas extended from southwestern Germany at the border with France along the valley of the Upper Rhine toward western Germany. The area represented 9,510 km^2^ of the country (2.7%). The EuroLST bioclimatic variable with the strongest influence on USUV risk was the annual mean temperature, contributing 71.4% to the variation in the ensemble of models. The next most influential variables were mean temperature of the coldest quarter of the year (8.9%), temperature seasonality (7.2%), and minimum temperature of the coldest month (5.2%). The other 5 variables had <5.0% effect each on USUV risk: mean temperature of the warmest quarter (2.8%), mean diurnal range (1.6%), temperature annual range (1.3%), maximum temperature of the warmest month (1.2%), and isothermality (0.4%) (Technical Appendix Figure 2). With an AUC value of 0.89 (±0.08 SD), 10-fold cross-validation indicated high predictive power of the BRT ensemble map.

**Figure 3:**
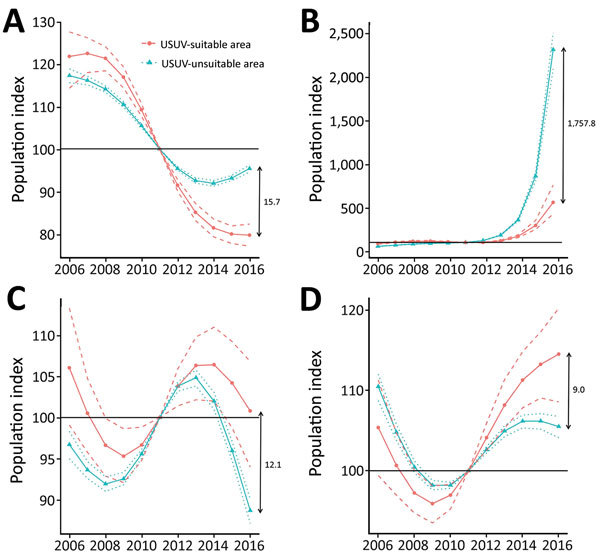
Index curves of the generalized additive model (GAM) approach with 300 bootstraps for breeding bird survey data of 4 bird species for Usutu virus (USUV)–suitable and USUV-unsuitable areas in Germany, 2016. A) Common blackbird; B) Eurasian tree sparrow; C) house sparrow; D) great tit. Solid lines indicate the mean indices from a GAM with 3 df; dashed/dotted lines represent nonoverlapping 95% bootstrap CIs. The horizontal line indicates the baseline year 2011 (index = 100), which is the last time point when bird abundance data were collected before the first known epizootic outbreak of USUV in Germany. Double arrows indicate the difference between the mean index curves for 2016.

Eleven of 15 bird species analyzed did not show a statistically significant difference in the population development between the areas suitable and unsuitable for USUV since 2011. We determined overlapping 95% bootstrap CIs between USUV-suitable and USUV-unsuitable areas for 2016 (Table; Technical Appendix Figure 3). Only 4 species had nonoverlapping CIs in 2016 (Table; Figure 3), that is, statistically significant different population indices between both areas. However, the great tit and house sparrow showed higher population indices in the USUV-suitable areas, indicating no negative population impact of USUV. The Eurasian tree sparrow had a statistically significant lower population index in the USUV-suitable area, but the species’ populations showed a very sharp positive development in both areas compared with the baseline year 2011. Thus, with a difference of ≈15.7% between the means of population indices in 2016, only the common blackbird showed both a statistically significant lower population index compared with the baseline year (CIs <100) and a statistically significant lower population index in the USUV-suitable area compared with the USUV-unsuitable area (nonoverlapping CIs between both areas).

**Table:** Bird population indices by species differentiated USUV-suitable and USUV-unsuitable areas, Germany, 2016*

Species	Mean population index, % (95% CI)		Difference in mean change between areas, %†
	USUV-suitable area	USUV-unsuitable area	
Eurasian blue tit (*Cyanistes caeruleus*)	115.0 (108.3–120.8)	107.5 (105.8–108.8)	7.5
Common chaffinch (*Fringilla coelebs*)	98.5 (93.0–104.4)	93.6 (91.8–95.4)	4.9
Eurasian magpie (*Pica pica*)	104.1 (98.4–109.9)	97.0 (95.5–98.4)	7.0
Eurasian tree sparrow (*Passer montanus*)	560.8 (428.4–760.6)	2,318.7 (2,097.0–2,511.5)	−1,757.8
European greenfinch (*Chloris chloris*)	78.2 (72.5–83.6)	76.1 (74.6–77.4)	2.1
Black redstart (*Phoenicurus ochruros*)	49.8 (46.0–53.2)	50.4 (49.5–51.3)	0.6
Common blackbird (*Turdus merula*)	79.7 (77.1–82.3)	95.4 (94.6–96.2)	−15.7
House sparrow (*Passer domesticus*)	100.8 (94.0–106.8)	88.7 (87.2–90.2)	12.1
Great tit (*Parus major*)	114.5 (108.6–120.2)	105.5 (104.1–106.8)	9.0
Common swift (*Apus apus*)	67.7 (59.6–76.7)	73.2 (70.9–75.9)	-5.6
Common house martin (*Delichon urbicum*)	74.8 (66.8–83.1)	73.9 (71.4–76.5)	0.9
Carrion crow (*Corvus corone*)	129.8 (107.4–155.2)	119.3 (113.8–123.6)	10.5
Common wood pigeon (*Columba palumbus*)	191.4 (172.2–212.6)	175.0 (170.6–180.0)	16.5
European robin (*Erithacus rubecula*)	101.1 (94.5–108.0)	97.8 (95.9–99.5)	3.3
Common starling (*Sturnus vulgaris*)	106.9 (98.9–115.1)	115.8 (112.8–118.9)	−8.8

*USUV, Usutu virus. †The difference in the mean change shows the magnitude and direction of divergence between the USUV-suitable and USUV-unsuitable area, i.e. a negative value indicates a lower population index for the USUV-suitable compared to the USUV-unsuitable area.

## Discussion

During the past 2 decades, an ongoing spread of USUV and a continuous circulation of the virus after initial establishment have been observed in different countries in Europe (*2**,**10*), highlighting the demand to understand the distribution of USUV and its ecosystem effects in the outbreak areas. Due to the enzootic transmission cycle of USUV with birds as amplifying hosts, the question regarding the effect of the virus on avian populations in particular was open. In this study, we compared the population dynamics of 15 common bird species between regions in Germany identified as USUV-suitable and USUV-unsuitable. Previous assessments were particularly limited by the missing explicit spatial distinction between areas with and without circulation of USUV. In addition, these studies did not distinguish population-level impacts of the disease from the natural fluctuations; that is, they did not use long-term bird abundance data that extend before and after the emergence of the first USUV outbreak.

Therefore, in a first step, we applied a distribution modeling approach to identify areas with and without potential circulation of USUV in Germany. The applied modeling approach was previously shown to be suitable to map the distribution of mosquitoborne viruses like dengue virus (*35*) or Zika virus (*28*). Potential outbreak areas for USUV were predominantly located in southwestern Germany, where the annual mean temperature was the most influential variable explaining the observed distribution (i.e., the virus showed an increasing probability of occurrence with increasing annual mean temperatures). Although the main transmission parameters of USUV are unknown (e.g., extrinsic incubation period), temperature is probably one of the most important drivers of USUV circulation, because the mosquito vectors are exothermic and the replication rate of the viruses increases as temperature increases. For example, laboratory experiments demonstrated that higher temperatures resulted in higher USUV infection rates of *Culex pipiens* mosquitoes (*36*). Nevertheless, although the ensemble of boosted regression tree models had a high performance in the differentiation of areas suitable and unsuitable for USUV in Germany, it should be kept in mind that this estimation has some degree of uncertainty. A high probability of occurrence for USUV does not necessarily mean that the virus ultimately arrives and establishes itself (*28*). In addition to environmental suitability, different additional parameters influence the spread and circulation of arboviruses, including vector/host mobility or herd immunity (*19**,**37*). 

Furthermore, the annual dynamics of USUV highly depend on the temporal temperature profile within the course of the year. The high activity of USUV in the late summer and beginning of autumn 2016, for example, was linked to temperature anomalies in September; significant positive deviation from the 30-year mean temperatures will have shortened the extrinsic incubation period and, at the same time, potentially caused increased vector abundance and associated vector-host contact rate (*12**,**19*).

Was observed statistically significant stronger decline of the population in the USUV outbreak areas compared with the USUV-unsuitable area only for the common blackbird and not for any other analyzed bird taxa, including species regularly tested positive for USUV in Europe (e.g., house sparrow and common starling) (*2*). This finding is in contrast to other mosquitoborne avian viruses, which often show negative effects on the populations of several bird species at the same time; WNV in North America negatively affected the populations of >7 bird species, leading to population reduction of up to 45% (*15*), and avian malaria parasites potentially caused the extinction of several bird taxa in Hawaii, USA (*16*). One possible explanation might be that population declines of some species are masked by natural population dynamics or spatial-temporal variability of the population fluctuations (*15*), such as those caused by largely neglected bird pathogens like polyomaviruses (*38*). Nevertheless, although USUV can infect >30 bird taxa, blackbirds are generally by far the most frequently affected species, comprising >60% of all bird specimens testing positive for USUV in Europe (*2*). The underlying causes of a remarkably higher frequency of USUV-positive common blackbirds compared with other bird species are unknown, but some factors may include the wide distribution and abundance of the species (*39*), its conspicuous size and color, and its close association with humans (*40*), all of which might contribute to the high recovery rate of blackbird bodies. Potential reasons for a higher sensitivity to USUV might be a higher virus susceptibility (*41*), behavioral traits (*42*), or different spatial-temporal distribution in relation to the vector/virus distribution (*43*).

During the USUV outbreak in Germany, the common blackbird population decreased by an additional 15.7% in the USUV-suitable area compared with the USUV-unsuitable area. Thus, assuming a mean density of 111.93 birds/km^2^ (8 million breeding pairs each having approximately 3 fledglings per year [*44*] and a USUV-suitable area of 9,510 km^2^), >167,119 birds died due to USUV since 2011; this estimate does not include other population effects like immigration compensating a part of the USUV-related population decline. The estimate is substantially higher than the one determined in the study by Bosch et al. (*21*), assuming 40,000 common blackbird deaths in the USUV outbreak in Germany in 2011–2012, which did not account for persistent USUV-related deaths. At the same time, the overall population decline of the common blackbird is considerably smaller than 50%–90% (*22**,**23*), which might reflect only the short-term population declines. Nevertheless, several studies reported local extinction of common blackbirds probably caused by the USUV outbreak (*18**,**21**,**22*), which can be explained by local high virus transmission (e.g., favorable distribution of vectors and hosts). A relatively large spatial heterogeneity of the impact of mosquitoborne viruses on bird populations was also observed for WNV in North America (*15*); that rate is potentially related to the connection between the local vector and the bird community and influenced by land use and climate parameters (*43*).

USUV activity after the first outbreak in Germany in 2011–2012 was remarkably lower in the following years, as reflected in the detection of fewer USUV-positive dead birds (*25**,**26*). However, the common blackbird population in the USUV-suitable areas continued to decline after the initial outbreak. A similar observation was made for WNV in North America, which has a persistent impact for different bird species resulting in a lower survival rate without signs of recovery after the first outbreak of the virus (*45*). Therefore, due to the ongoing and widespread circulation of USUV in Central Europe (*12*), we must expect a long-term decline of common blackbird populations in areas with USUV occurrence, leading to a substantial alteration of the bird communities in the European USUV outbreak areas.

We still lack comprehensive data on the interaction between USUV and its vectors, hosts, and environmental parameters. We need data on the avian hosts to clarify the epidemiology of USUV and investigation into ecologic consequences, especially if they increase the risk of infection for humans. For example, the high mortality rate among common blackbirds might increase the chance of USUV spillover to humans, because the dead hosts are not present as immune or dead-end hosts (*15**,**43**,**46**,**47*). In addition, although no USUV effect was found for bird species classified as threatened (e.g., common starling) (*48*), a wide variety of bird species may be susceptible to USUV infections (*2*). Therefore, further studies should also focus on bird species not covered by the bird abundance dataset used here, such as wetland birds, which occupy areas that generally harbor high numbers of mosquitoes (*49*).

In summary, USUV had a statistically significant negative impact on the population of common blackbirds in suitable areas in Germany: a lower population index compared with the baseline year (CIs <100) and a statistically significant lower population index in the USUV-suitable area compared with the USUV-unsuitable area (nonoverlapping CIs between both areas). We observed no significant effect for the other 14 bird species included in the study. Five years after the first detection of USUV in southwest Germany, the circulation of the virus resulted in an additional decline of ≈15.7% in the common blackbird populations compared with the development of populations not affected by USUV. Avian populations are under different threats, including changes of land use and climate change (*15**,**24*). The emergence of USUV in Europe is a further threat that can cause substantial changes in ecosystem services provided by birds, such as seed dispersal (*50*). The recent outbreak of USUV in 4 Central European countries (Germany, Netherlands, Belgium, and France) underlines the large-scale distribution of USUV spanning from southern to central Europe (*2**,**12*). In conclusion, USUV could affect bird populations, at least common blackbirds, across Europe.

Technical Appendix Additional information regarding the analysis of Usutu virus in Germany.
