# Supplementary material for: Distribution of Usutu Virus in Germany and Its Effect on Breeding Bird Populations
Source: Emerg Infect Dis. 2017 Dec;23(12):1994–2001. doi: 10.3201/eid2312.171257 (PMC5708248; doi:10.3201/eid2312.171257)
Supplement: Technical Appendix — Additional information regarding the analysis of Usutu virus in Germany. [file 17-1257-Techapp-s1.pdf]

Article DOI: https://doi.org/10.3201/eid2312.171257

Distribution of Usutu Virus in Germany and Its Effect on Breeding Bird Populations

Technical Appendix

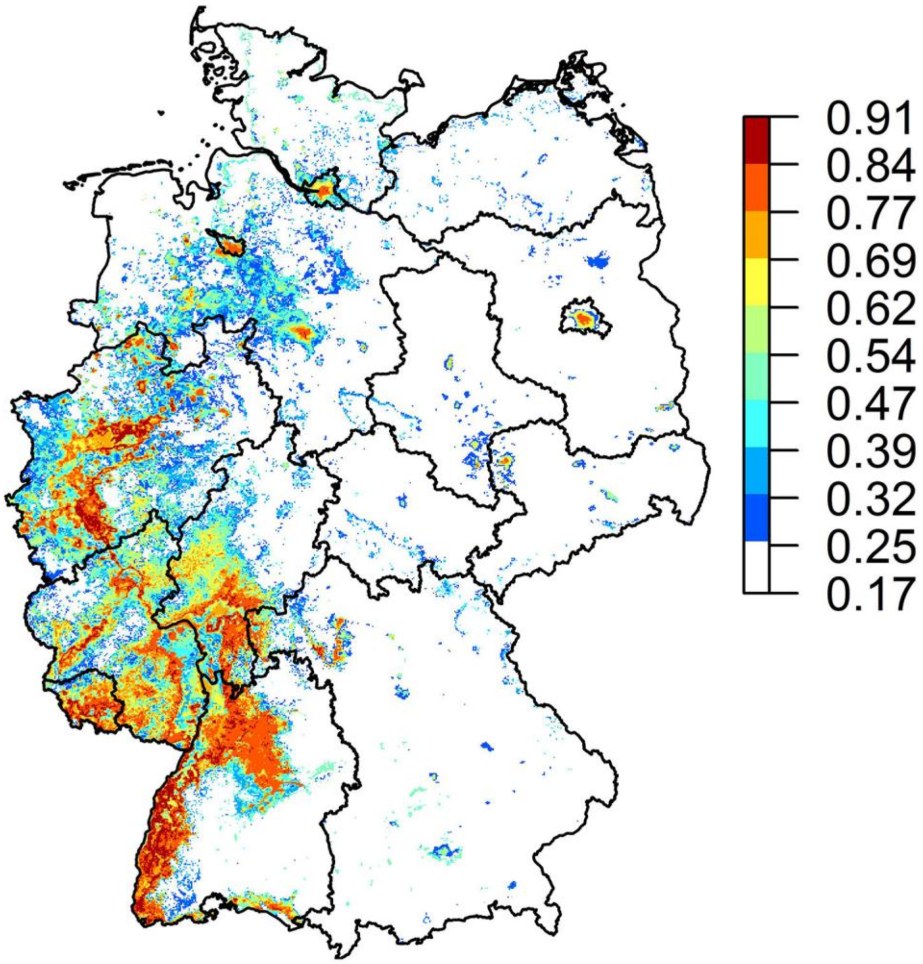

Technical Appendix Figure 1. Uncertainty (size of the 95% confidence interval) around the ensemble of 300 boosted regression tree models for the prediction of Usutu virus in Germany.

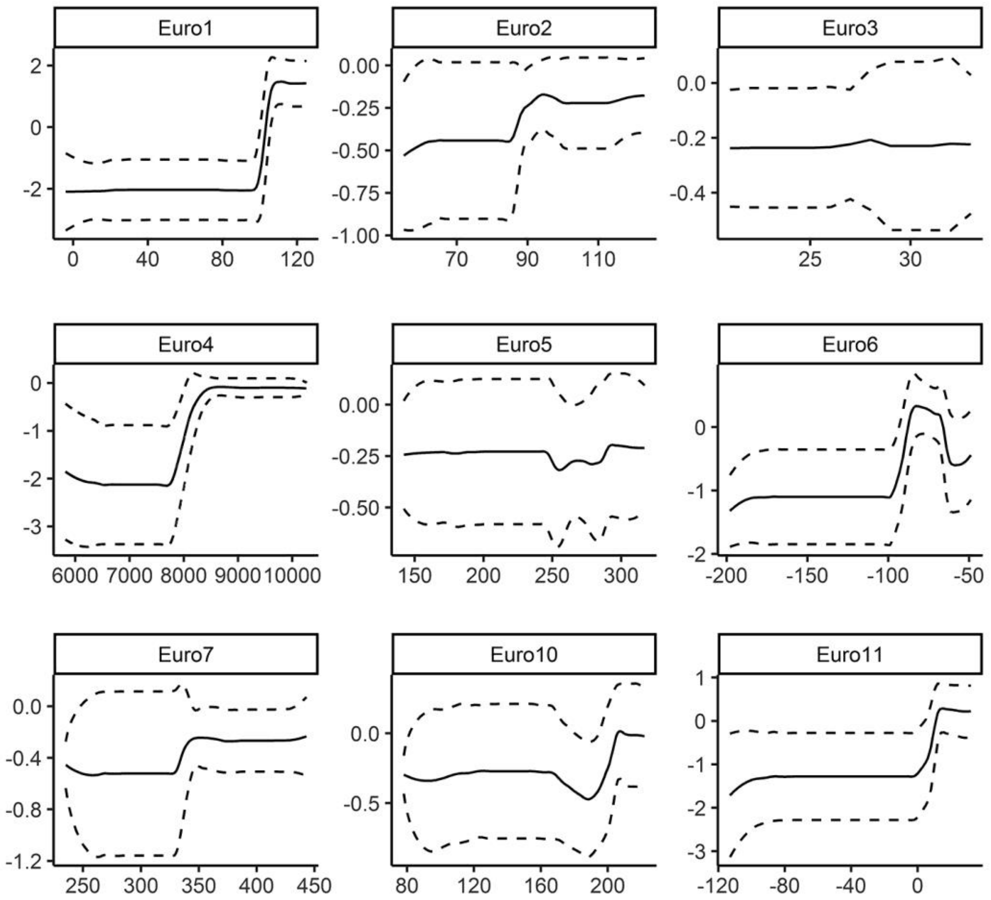

Technical Appendix Figure 2. Effect plots for each covariate from the European Land Surface Temperature variables in the ensemble of 300 BRT for the prediction of Usutu virus in Germany. The solid line gives the mean and the dashed lines represent upper and lower 95% bootstrapped confidence limits. Euro1, annual mean temperature (°C*10); Euro2, mean diurnal range (mean monthly (max – min temp)); Euro3, isothermality ((bio2/bio7)*100); Euro4, temperature seasonality (standard deviation * 100); Euro5, maximum temperature of the warmest month (°C*10); Euro6, minimum temperature of the coldest month (°C*10); Euro7, temperature annual range (bio5 – bio6) (°C*10); Euro10, mean temperature of the warmest quarter (°C*10); Euro11, mean temperature of the coldest quarter (°C*10).

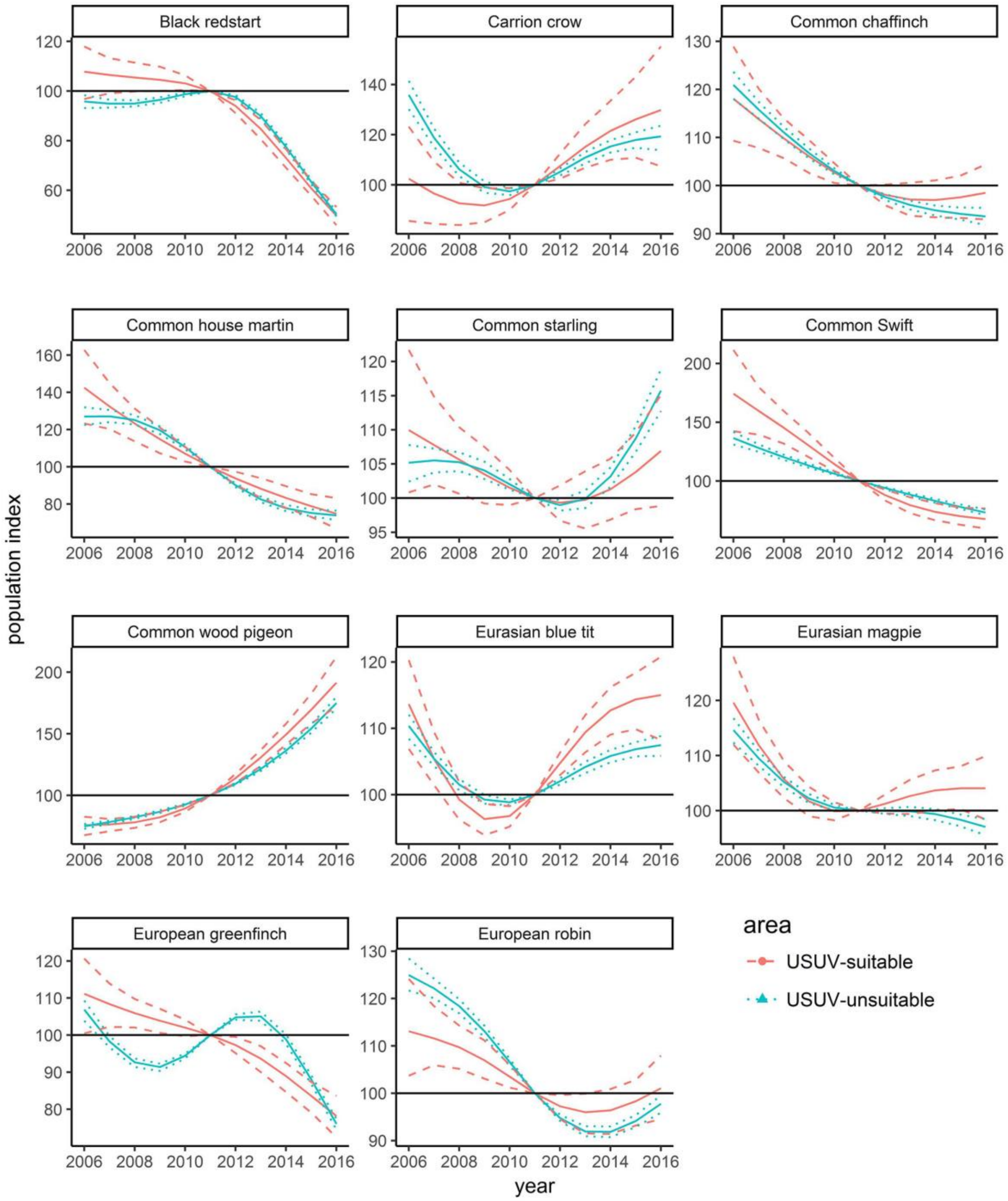

Technical Appendix Figure 3. Index curves of the General Additive Model (GAM) approach with 300 bootstraps for breeding bird survey data of 11 bird species with overlapping 95% bootstrap CI between the USUV-suitable and USUV-unsuitable areas. Solid lines give the mean indices from a GAM with 3 df and the dashed/dotted lines represent upper and lower 95% bootstrapped confidence limits. The horizontal line indicates the baseline year 2011 (index = 100), which is the last time point when bird abundance data were collected before the first epizootic outbreak of USUV in Germany.
